# Association between mitochondrial DNA copy number and cardiovascular disease: Current evidence based on a systematic review and meta-analysis

**DOI:** 10.1371/journal.pone.0206003

**Published:** 2018-11-07

**Authors:** Peng Yue, Siyuan Jing, Lei Liu, Fan Ma, Yi Zhang, Chuan Wang, Hongyu Duan, Kaiyu Zhou, Yimin Hua, Gang Wu, Yifei Li

**Affiliations:** 1 Department of Pediatrics, West China Second University Hospital, Sichuan University, Chengdu, Sichuan, China; 2 Ministry of Education Key Laboratory of Women and Children's Diseases and Birth Defects, West China Second University Hospital, Sichuan University, Chengdu, Sichuan, China; 3 West China Medical School, Sichuan University, Chengdu, Sichuan, China; 4 Program for Changjiang Scholars and Innovative Research Team in University, West China Second University Hospital, Sichuan University, Chengdu, Sichuan, China; University of Mississippi Medical Center, UNITED STATES

## Abstract

**Background:**

Mitochondria are energy-producing structure of the cell and help to maintain redox environment. In cardiovascular disease, the number of mitochondrial DNA (mtDNA) will changes accordingly compare to normal condition. Some investigators ask whether it has a clear association between mtDNA and cardiovascular disease with its adverse events. Thus, we conduct the meta-analysis to assess the role of circulating mtDNA in evaluating cardiovascular disease.

**Methods:**

The meta-analysis was conducted in accordance with a predetermined protocol following the recommendations of Cochrane Handbook of Systematic Reviews. We searched the Pubmed, Embase, the Cochrane Central Register of Controlled Trials and World Health Organization clinical trials registry center to identify relevant studies up to the end of October 2017. Data were analyzed using STATA. Besides, publication bias and meta-regression analysis were also conducted.

**Results:**

We collected results from 5 articles for further analyses with 8,252 cases and 20,904 control. The normalized mtDNA copy number level is lower in cardiovascular disease (CVD) than the control groups with a pooled standard mean difference (SMD) of -0.36(95%CI,-0.65 to -0.08); The pooled odds ratio (OR) for CVD proportion associated with a 1-SD (standard deviation) decrease in mtDNA copy number level is 1.23 (95% CI,1.06–1.42); The OR for CVD patients with mtDNA copy number lower than median level is 1.88(95% CI,1.65–2.13); The OR for CVD patients with mtDNA copy number located in the lowest quartile part is 2.15(95% CI, 1.46–3.18); the OR between mtDNA copy number and the risk of sudden cardiac death (SCD) is 1.83(95% CI, 1.22–2.74).

**Conclusion:**

Although inter-study variability, the overall performance test of mtDNA for evaluating CVD and SCD revealed that the mtDNA copy number presented the potential to be a biomarker for CVD and SCD prediction. Given that, the fewer copies of mtDNA, the higher the risk of CVD.

## 1. Introduction

Cardiovascular diseases (CVDs), especially coronary vascular disease, ischemic heart failure, and cardiomyopathy, are major causes of clinical mortality and lead to a significant health and economic burden worldwide[1]. Decades have passed since researchers started to identify diagnostic biomarkers for CVDs to predict the disease prognosis. However, although some parameters have been identified, most of them, such as brain natriuretic peptide (BNP) and N-terminal portion of proBNP (NT-proBNP), are focused on the acute process of CVDs. With the advantages of these parameters, the diagnosis of specific diseases can be reached; unfortunately, they can only provide limited information about cardiomyocyte function and prognosis understanding [2]. Accordingly, finding a new and highly specific biomarker to reveal real cardiac function and predict the prognosis of CVDs is an emerging demand.

Mitochondria are double-membrane structures that exist in all cells. They are the energy-producing structures of the cell. Mitochondria are especially important in cardiomyocytes, because they account for 40% of the cardiac cell volume, and 90% of the energy required for the heart is supplied by mitochondria. Mitochondrial DNA (mtDNA) is a kind of double-stranded DNA molecule, encoding 2 ribosomal RNAs, 22 transfer RNAs, and 13 polypeptides of the respiratory chain [3, 4]. Different from nuclear DNA, mtDNA is vulnerable to reactive oxygen species (ROS) damage resulting from the lack of histone protection and effective DNA repair mechanisms [5, 6]. Once mtDNA damage occurs, the copy number level of mtDNA is altered, resulting in mitochondrial dysfunction that is considered to be an important pathogenesis of CVDs [7]. The mtDNA copy number could reflect the level of mtDNA damage; it is thought to be an indicator of mitochondrial function [8]. Therefore, some researchers hypothesize that we can predict the risk and prognosis of CVDs by measuring the copy numbers of mtDNA. However, there is no general consensus on this hypothesis. Thus, we performed a meta-analysis to determine whether changes in the mtDNA copy number are related to the severity of CVDs and their prognoses.

## Materials and methods

### Study protocol

The analysis was performed following the recommendations of the Cochrane Handbook of Systematic Reviews. Data collection and reporting strictly followed the PRISMA Statement. Furthermore, all participants received training by the Cochrane Center of China on how to conduct a systematic review and meta-analysis.

### Search strategy

PubMed, Embase, the Cochrane Central Register of Controlled Trials and the World Health Organization International Clinical Trials Registry Platform were searched using a strategy to obtain the publications for review as “((((copy number) AND ((mitochondrial [MeSH Terms] AND DNA) OR mtDNA OR mitochondrial DNA))) AND ((heart OR cardiology OR cardiac OR cardiology [MeSH Terms] OR heart [MeSH Terms] OR cardiovascular [MeSH Terms]))”. The search included articles through October 31, 2017.

### Study selection

The inclusion criteria were as follows: 1) case-control or cohort study design; 2) the association level of mtDNA copy number and CVD risk was evaluated based on a case-control or cohort study; 3) studies performed on human beings; 4) total DNA was extracted from a circulating blood sample; 5) mtDNA copy numbers were measured by previously described methods such as qPCR or DNA sequence array; 6) all data were presented as odds ratios (ORs) and their 95% confidence intervals (CIs) or could be converted from the available data for this meta-analysis; 7) studies that clearly described how to identify the low level mtDNA copy number and its standard; 8) the data in the publication were sufficient.

Studies were excluded if any of the following applied: 1) repeat publications, abstracts, letters or reviews; 2) research failed to obtain an adjusted OR to reduce the bias; 3) the mtDNA copy number was used to differentiate types of CVD without a sufficient control population or cohort; 4) research focused on cerebral vascular diseases.

### Data collection and assessment of study quality

Two investigators (Peng Yue, Siyuan Jing) first independently assessed the eligibility of reports at the title and/or at abstract level in a blinded fashion, with a third reviewer (Yifei Li) determining the divergences; studies that met the inclusion criteria were selected for further analysis. The second step to evaluate the whole article could not be conducted in a blinded fashion. All the baseline data from the included studies were extracted and are shown in Table 1. The Newcastle-Ottawa Scale (NOS) for assessing the quality of nonrandomized studies in meta-analyses was used to evaluate all included studies. The quality of enrolled studies was evaluated based on three areas: selection, comparability and outcome. This meta-analysis included studies that were deemed to have a moderate to high methodological quality; studies that scored at least 5 stars[9].

**Table 1:** Summary of included studies.

No.	Author	Year	Disease	Region	Samples	Methods for mtDNA level	Population		Design	Age(years)/Gender(% male)
							Case	Control		
1	_Huang_	_2016_	_HF_	_China_	_Blood/Leukocytes_	qPCR on mt-ND1 and HBB genes	1700	1700	Prospective, case-control study	57.9±13.4/65.6%
2	_Ashar ARIC_	_2017_	_CVD_	_UK_	_Blood/Leukocytes_	Affymetrix Genome-Wide Human SNP Array 6.07	1500	8650	Prospective, cohort study	57.9(45–65)/45.3% of whole participants
3	_Ashar CHS_	_2017_	_CVD_	_UK_	_Blood/Leukocytes_	TaqMan-based qPCR	1743	2383	Prospective, cohort study	72.5/45.3% of whole participants
4	_Ashar MESA_	_2017_	_CVD_	_UK_	_Blood/Leukocytes_	Affymetrix Genome-Wide Human SNP Array 6.07	422	5465	Prospective, cohort study	62.4(45–85)/ 45.3% of whole participants
3	_Zhang_	_2017_	_CVD_	_UK_	_Blood/Leukocytes_	Affymetrix 6.0 array	2219	2218	Prospective, cohort study	57.9±6.0/44.8%
4	_Chen_	_2014_	_CVD_	_China_	_Blood/Leukocytes_	qPCR on mt-ND1 and HGB genes	378	378	Prospective, case-control study	58.9±9.8/73.8%
5	_Liu_	_2017_	_CVD_	_China_	_Blood/Leukocytes_	qPCR on mt-ND1 and HGB genes	290	110	Prospective, cohort study	58.8±11.2/85.1%

### Evaluation indicators

The normalized mtDNA copy number level between the CVD and control groups was evaluated. In addition, the association between different mtDNA copy number thresholds and the risks of CVD and sudden cardiac death (SCD) were investigated. First, we used the mtDNA copy number as a continuous variable and evaluated the changes in risks of study outcomes that were associated with a 1-SD decrease in the mtDNA copy number. Two types of thresholds were used to separate high and low mtDNA copy numbers: a lower than median level, and the lowest quartile proportion that would help identify the power of the circulating blood mtDNA copy number to predict CVD and adverse events at different levels. The relative OR with a 95% CI was used.

### Publication bias

Publication bias was tested using funnel plots and Egger’s test with Stata statistical software (STATA) version 14.0. An asymmetric distribution of data points in the funnel plot and a quantified result of P< 0.05 in the Egger’s test indicated the presence of potential publication bias[10]. Asymmetry in the funnel plot was due to a variety of factors aside from publication bias, including small study effects, heterogeneity and chance, especially in studies with a small sample size.

### Heterogeneity

The χ^2^ test was used to examine heterogeneity in pooling sensitivity and specificity. Heterogeneity was considered to be statistically significant when P<0.05 in these qualitative tests. The I^2^ test was also conducted in every pooling analysis to quantitatively estimate the proportion of total variation across studies that was attributable to heterogeneity rather than chance. The I^2^ value ranges from 0 to 100%, with a value greater than 50% indicating significant heterogeneity.

### Sensitivity analysis

To determine whether any single study incurred undue weight in the analysis, a sensitivity analysis was conducted for every study using STATA 14.0 for the meta-analysis fixed/random-effects estimates.

### Statistical analysis

Data were analyzed using STATA Version 14.0[11]. Additionally, publication bias and a meta-regression analysis were conducted using STATA version 14.0. If there was an obvious heterogeneity among the studies (I^2^ > 50%), the random-effects model was used for the meta-analysis. Otherwise, the fixed-effects model was used.

## 3. Results

### 3.1. Study evaluation

**Fig 1:**
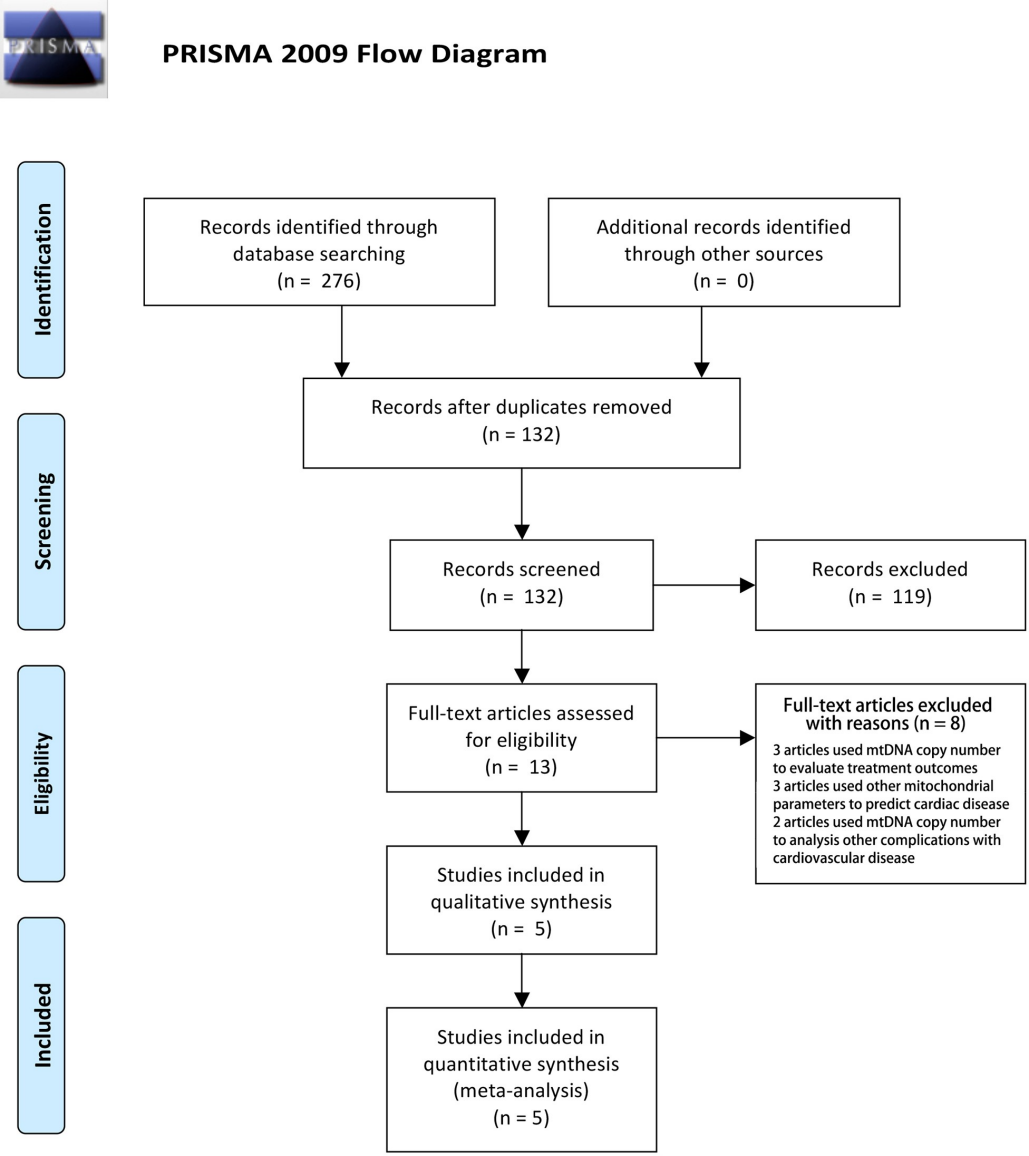
PRISMA flow chart of studies inclusion and exclusion. From: Moher D, Liberati A, Tetzlaff J, Altman DG, The PRISMA Group (2009). Preferred Reporting Items for Systematic Reviews and Meta-Analyses: The PRISMA Statement. PLoS Med 6(7): e1000097. doi:10.1371/journal.pmed1000097. For more information, visit http://www.prisma-statement.org.

A total of 132 citations were retrieved. After reading the titles and abstracts, 119 citations were excluded according to the selection criteria; initially, 13 articles were identified [2] [8, 12–22]. Among them, 8 articles were excluded after reading the whole article; in four of these, useful data was unable to be extracted for the meta-analysis, 2 articles were not focused on the relationship between CVD and the mtDNA copy number, and 2 articles used the mtDNA copy number to differentiate types of CVD without a sufficient control population or cohort. No articles were added through a manual retrospective search after reading the related publications. Finally, 5 articles with 7 studies of the association between the mtDNA copy number and CVD were enrolled into our meta-analysis (Fig 1). Particularly, one article included three studies: the Cardiovascular Health Study (CHS), the Atherosclerosis Risk in Communities Study (ARIC), and the Multiethnic Study of Atherosclerosis (MESA). The basic characteristics of the included studies are presented in Table 1. The PRISMA checklist was attached as S1 Table. All the excluded articles are listed in S1 File.

### 3.2 Publication bias

**Fig 2:**
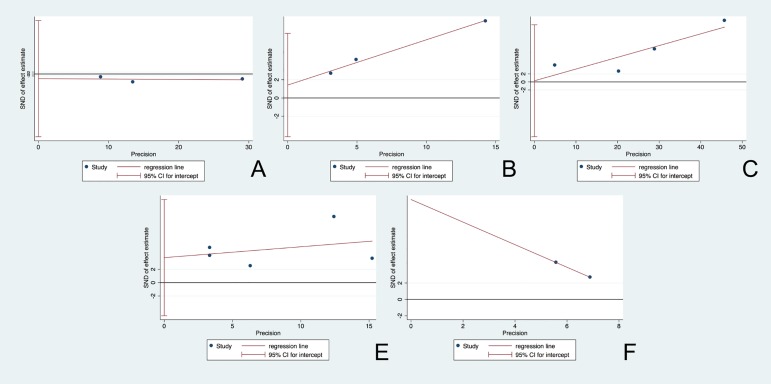
Funnel plot for the assessment of potential publication bias. The funnel graphs plot the square root of the effective sample size (1/ESS1/2) against the OR. Each dot represents each study in the meta-analysis. Asymmetry of the dot distribution between regression lines indicates potential publication bias. (A) for the evaluation of average level of mtDNA copy number, (B) for the risk analysis among the group below median mtDNA copy number level, (C) for the evaluation of risk increase by every 1-SD decrease in mtDNA copy number level, (D) for the risk measurement of the population located in the lowest part of quartile division of mtDNA copy number level, and (E) for the association analysis between mtDNA copy number and the risk of SCD. This funnel plot indicates no publication bias with a p value >0.05. OR = odds ratio, ESS = effective sample size.

To evaluate the publication bias of included studies, funnel plots were used (Fig 2). The absence of any asymmetric distribution suggested that there was no publication bias. An asymmetric distribution indicated there might be publication bias. According to the Egger’s test, the qualitative analysis indicated the absence of publication bias among all enrolled studies, for the mtDNA copy number, p = 0.495, t = -1.02, 95% CI (-63.23, 53.87); for the risk in every 1-SD decrease in mtDNA copy number, p = 0.937, t = 0.09, 95% CI (-13.73, 14.31); for the risk in lower than median mtDNA level, p = 0.194, t = 3.18, 95% CI (-4.26, 7.10); and for the risk in the lowest quartile, p = 0.264, t = 1.37, 95% CI (-4.95, 12.44). For SCD, only two studies were included, so a qualitative analysis for publication bias was unavailable. Based on the above results from the funnel plot assessment and Egger’s test, it was difficult to reach a conclusion on the existing publication bias, as the asymmetric triangle that might be followed included a small sample size and heterogeneity.

### 3.3 Average level of mtDNA copy number

**Fig 3:**
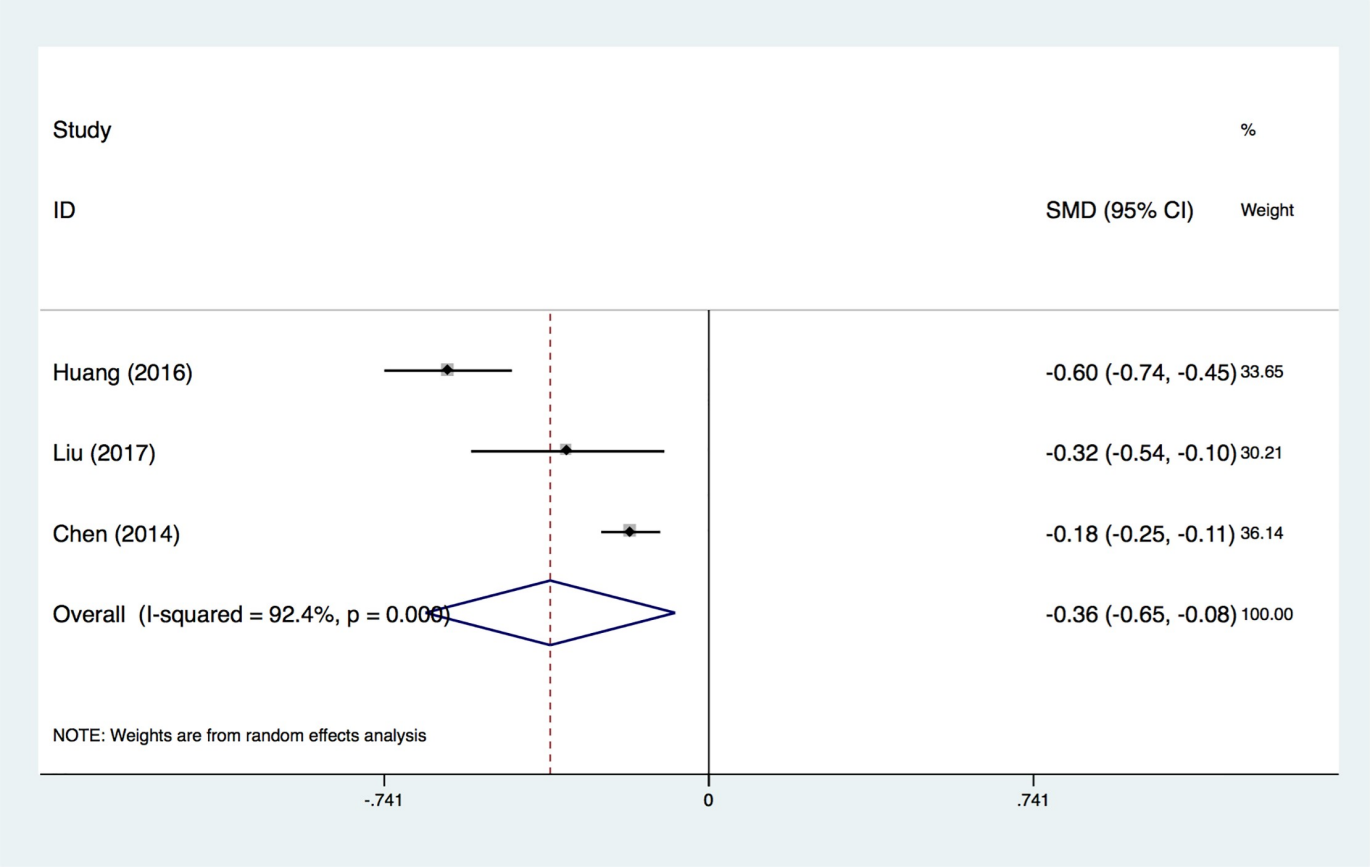
Forest plot for comparison of the normalized mtDNA copy number level between CVD and control groups. Only the first author of each study is given. SMD, the mean standard deviation.

In Fig 3, we pooled data from three independent studies to evaluate the normalized mtDNA copy number level between CVD cases and the control group. There was a significantly lower copy number of mtDNA in circulating peripheral blood among the CVD cases when compared with the control group (SMD = -0.36±0.28) (Fig 3). Based on results from the random-effects analysis, there was heterogeneity across the studies (I^2^ = 92.4%, P<0.0001).

### 3.4 Association between mtDNA copy number level and CVD risks

**Fig 4:**
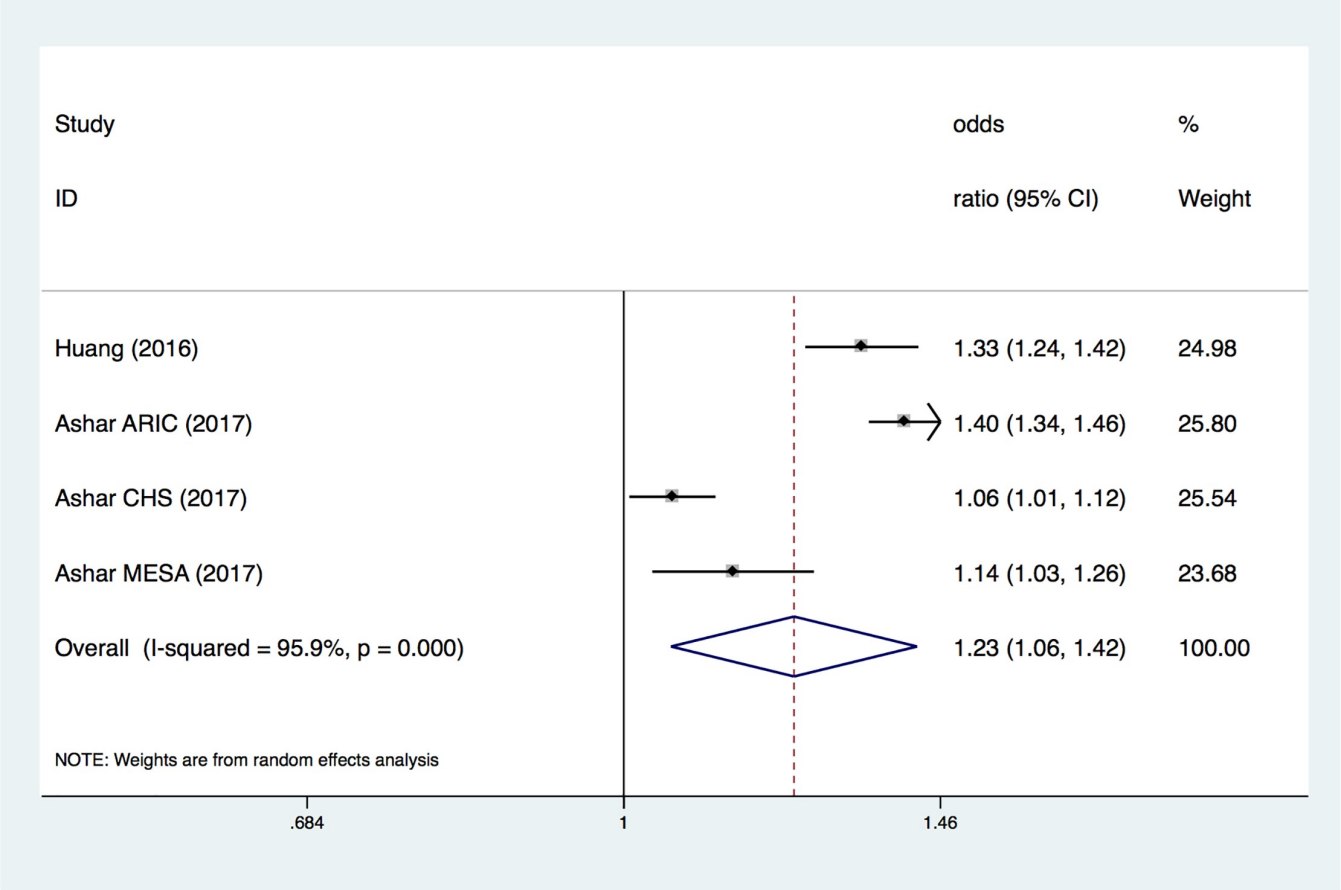
ORs for CVD associated with every 1-SD below in mtDNA copy number measurement. Only the first author of each study is given. CI, confidence interval.

The overall OR for every 1-SD below the mtDNA copy number measurement (Fig 4) showed an increased risk of mtDNA copy number in CVD (OR, 1.23; 95% CI, 1.06–1.42). Based on the results of the random-effects analysis, there was heterogeneity across the studies (I^2^ = 95.9%, P<0.0001) (Fig 4).

**Fig 5:**
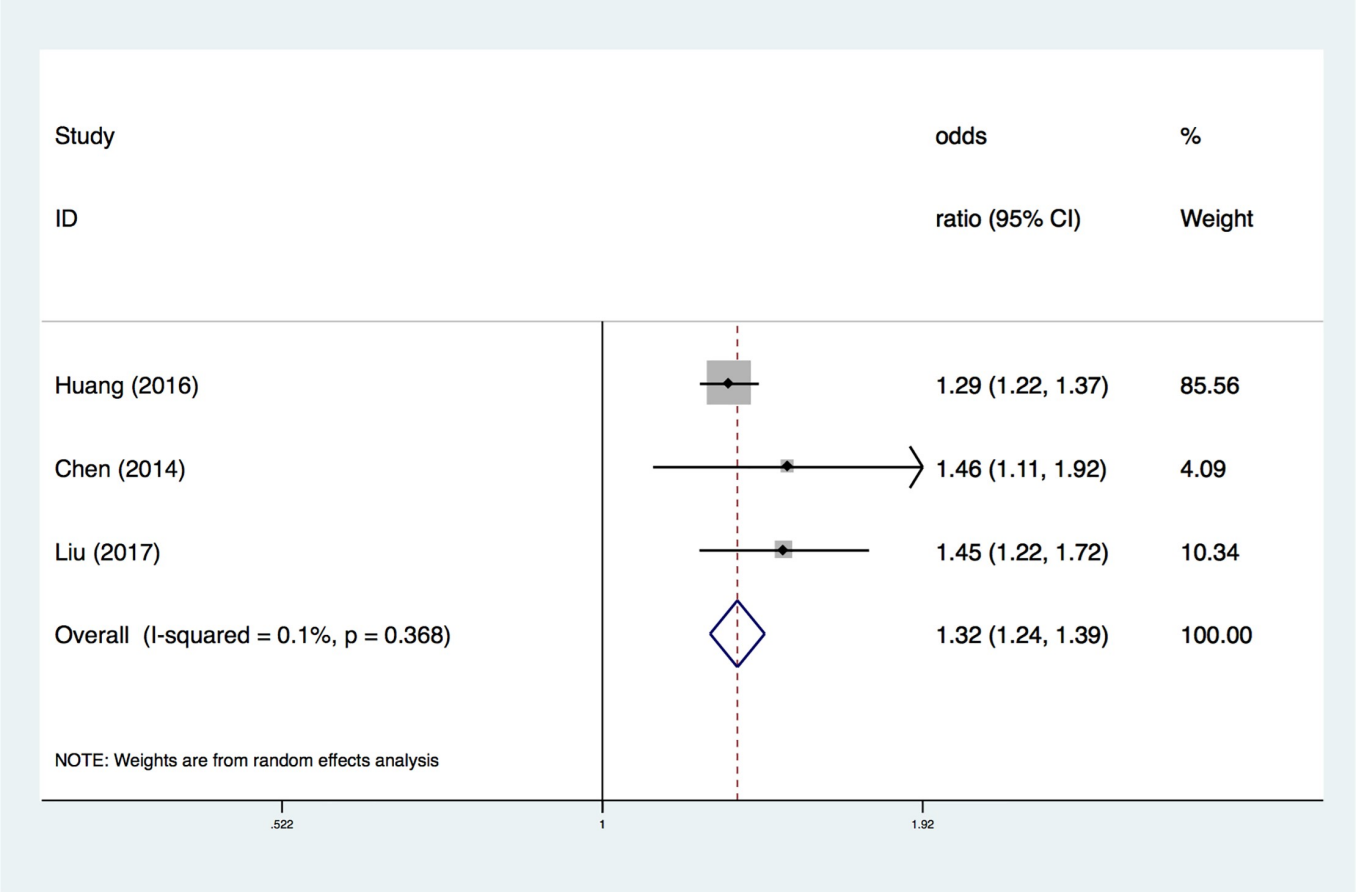
ORs of CVD among the mtDNA copy number lower than median level proportion. Only the first author of each study is given. CI, confidence interval.

The overall OR from the proportion of the mtDNA copy number lower than the median level of the total participants (Fig 5) confirmed that the risk increased as the mtDNA level decreased (adjusted OR, 1.32; 95% CI, 1.23–1.39). Although there was no heterogeneity across the studies (I^2^ = 0.1%, P = 0.368), the results were also conducted using a random-effects analysis according to their complicated sample collections (Fig 5).

**Fig 6:**
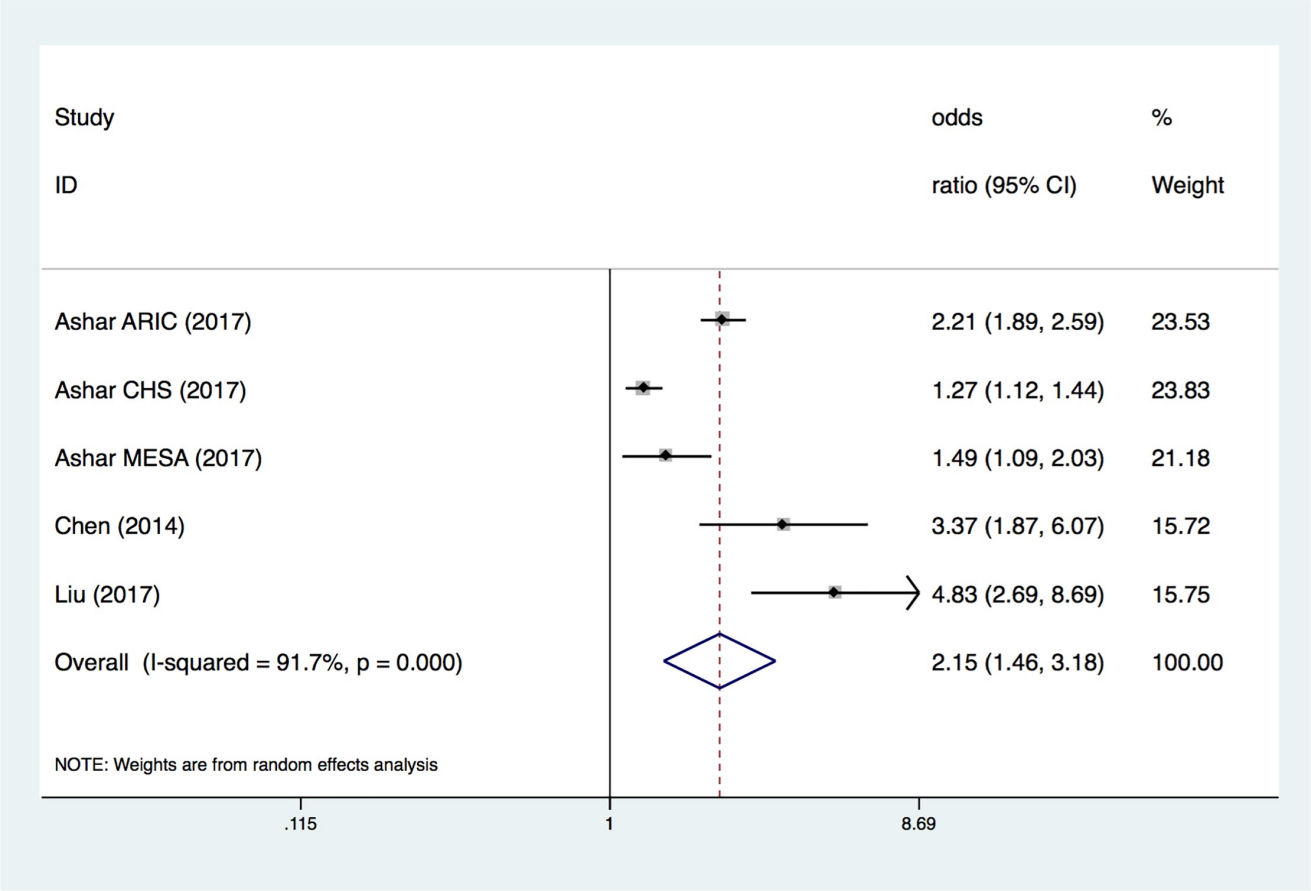
ORs of the population located in lowest quartile level in mtDNA copy number. Only the first author of each study is given. CI, confidence interval.

The overall OR of the population located in the lowest quartile of mtDNA copy number (Fig 6) demonstrated the highest risk in suffering from CVD (adjusted OR, 2.15; 95% CI, 1.46–3.18). There was heterogeneity across the studies (I^2^ = 91.7%, P<0.0001), based on results from the random-effects analysis (Fig 6).

### 3.5 Association between mtDNA copy number and SCD

**Fig 7:**
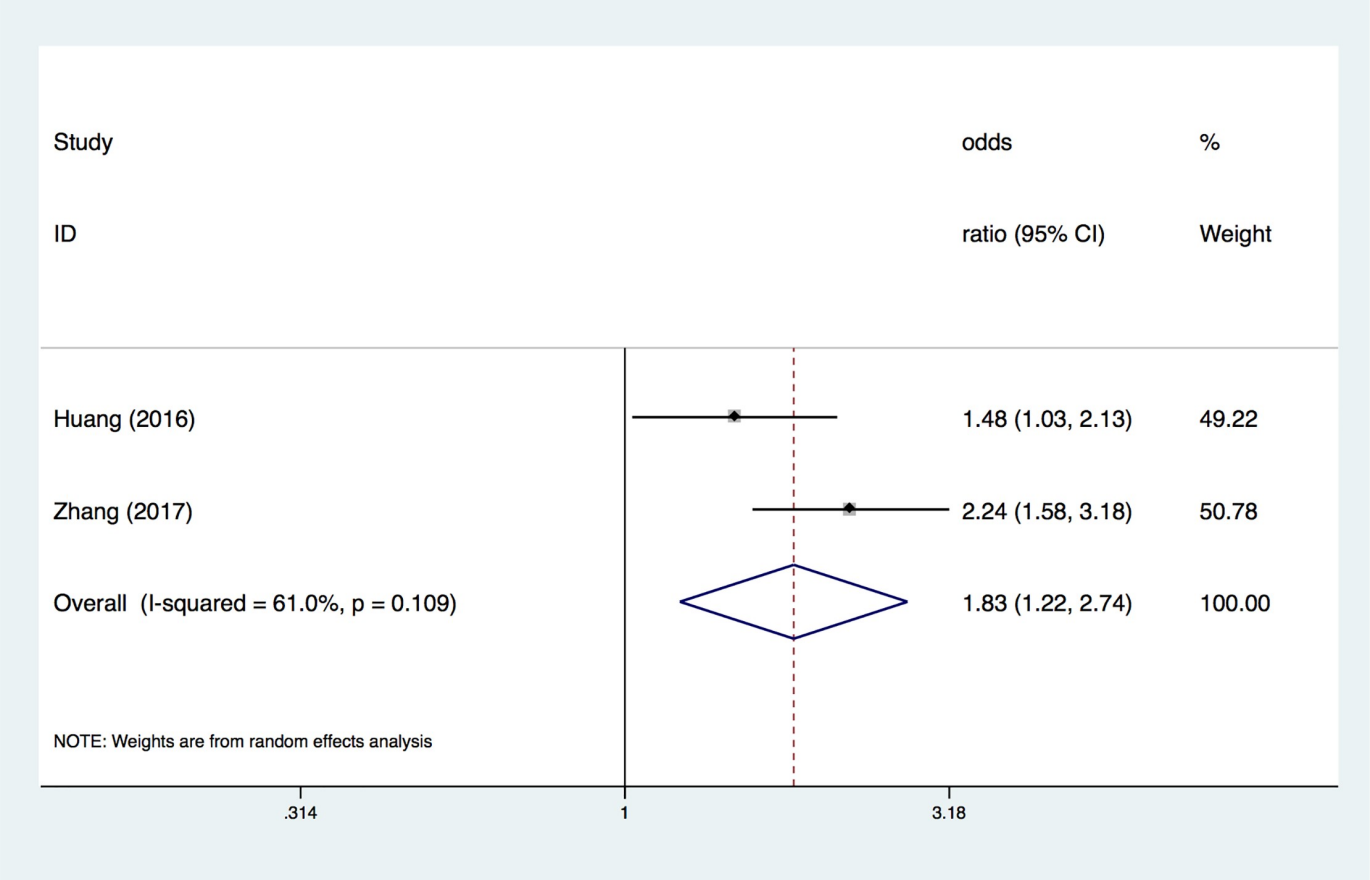
ORs for the association between mtDNA copy number and SCD. Only the first author of each study is given. CI, confidence interval.

The overall OR of the association between the mtDNA copy number and SCD (Fig 7) identified the relationship between mtDNA copy number and SCD (adjusted OR, 1.83; 95% CI, 1.22–2.74). There was heterogeneity across the studies (I^2^ = 61%, P = 0.109), based on results from the random-effects analysis (Fig 7).

### 3.6 Sensitivity analysis

**Fig 8:**
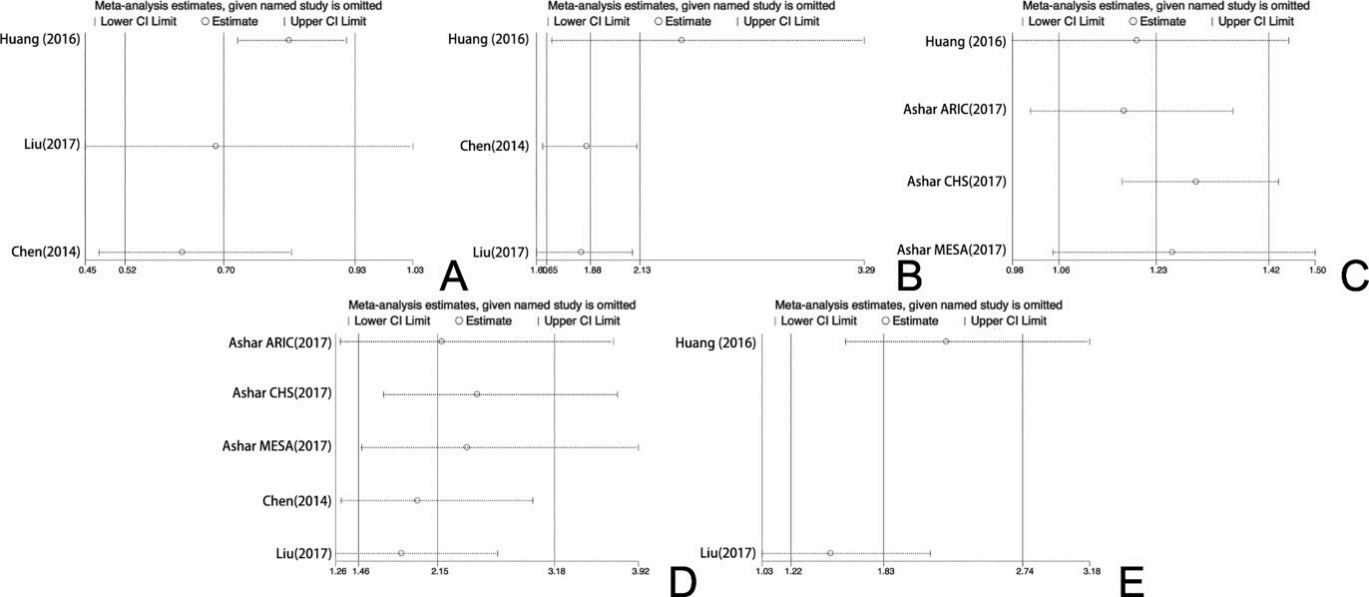
In each diagram, the vertical solid line in the middle represents the total merge effect, and the two vertical solid lines on the left and right represent the upper and lower limits of the 95% confidence interval of the total consolidation effect; The cross line corresponding to each study represents the combined effect of the remaining studies after the deletion of the study.

We systematically and qualitatively analyzed the sensitivity across the included studies (Fig 8). The results of Figures A, B, C, D and E indicate that no single data set carried enough weight to significantly influence the pooled test performance reported for the ability of the mtDNA copy number to predict CVD and SCD. Finally, the sensitivity analysis was double checked by removing one data set at a time; the analysis confirmed both the direction and magnitude of the statistical significance of the findings from the overall analysis.

## 4. Discussion

The meta-analysis included a total of seven studies in five articles [2, 8, 16, 21, 22]; these were selected in strict accordance with the inclusion and exclusion criteria. A publication bias analysis and sensitivity analysis of each study was performed. We conducted a systematic evaluation of each study using the NOS for quality assessment, and the scores were acceptable (Table 2). To our knowledge, our meta-analysis was the first meta-analysis conducted on the association between different mtDNA copy number thresholds and the risks of CVD and SCD. Therefore, our research provides more convincing evidence in the field.

**Table 2:** Summary of Newcastle-Ottawa Scale scores in quality evaluation.

No.	Author	Year	Selection	Comparability	Outcome	Total NOS scores	Methods to reduce bias
1	_Huang_	_2016_	4	2	2	8	Adjusted for age, sex, smoking status, hypertension, hyperlipidemia, and diabetes
2	_Ashar_	_2017_	4	2	3	9	Adjusted for age, sex, collection center, and race/ethnicity
3	_Zhang_	_2017_	4	2	3	9	Adjusted for age, race, sex, and enrolment centre
4	_Chen_	_2014_	3	2	3	9	Adjusted for individual traditional CHD risk factors, including age, sex, smoking and drinking status, drug intake, history of hypertension and diabetes mellitus, SBP, DBP, TC, TG, HDL-C and LDL- C
5	_Liu_	_2017_	3	2	3	8	Adjusted for individual traditional CHD risk factors, including age, sex, smoking and drinking status, drug intake, history of hypertension and diabetes mellitus, SBP, DBP, TC, TG, HDL-C and LDL- C

In our meta-analysis, we conducted three pooled evaluations: 1) to evaluate the normalized mtDNA copy number level between CVD and control groups; 2) to evaluate the association between different mtDNA copy number thresholds and the risk of CVD; and 3) to evaluate the association between the mtDNA copy number and the risk of SCD. In the first pooled evaluation, the pooled SMD was -0.36 (95% CI, -0.65 to -0.08), indicating that detecting the mtDNA copy number was lower in CVD patients than in the control group. Although there was heterogeneity across the studies (I^2^ = 92.4%, P<0.0001), the sensitivity analysis showed that no single data set carried enough weight to significantly influence the pooled test performance. According to the heterogeneity measurement, we considered that the heterogeneity of the overall OR analysis of every 1-SD below the mtDNA copy number measurement and CVD came from a single study and demonstrated a large range for the 95% CI. Furthermore, the heterogeneity of the overall OR analysis of the population located in the lowest quartile of mtDNA copy number and CVD could be due to only two studies being recruited. Thus, we consider that the results are reasonable and acceptable. Therefore, this result suggests that using the level of the mtDNA copy number could be used as a parameter to distinguish CVD patients from normal people.

Next, we investigated the specific relationship between CVD and the mtDNA copy number. We hypothesized that the lower a patient's copy number of mtDNA, the higher his risk of CVD. In the second pooled evaluation, we conducted three types of analyses to gain insight into the risk of CVD in different cohorts with several different mtDNA copy number thresholds. Using the mtDNA copy number as a continuous variable and evaluating the change in the risk of study outcomes associated with a 1-SD decrease in the mtDNA copy number revealed that the pooled OR was 1.23 (95% CI, 1.06–1.42). The pooled OR for CVD lower than the median level proportion was 1.88 (95% CI, 1.65–2.13); in the population located in the lowest quartile of mtDNA copy number, the combined OR increased to 2.15 (95% CI, 1.46–3.18). According to our results, there was a negative correlation between the mtDNA copy number and CVD risk. A small number of combined analyses showed that the decrease of mtDNA copy number in peripheral blood cells was associated with a higher risk of SCD, and the pooled OR was 1.83 (95% CI, 1.22–2.74).

Mitochondria are thought to be an endosymbiont of ancient bacteria permanently colonized in eukaryotes[23]. The genes they carry are evolved and integrated into the host genome. In humans, mitochondria retain only 37 genes[24]; most of the proteins translated by these genes are involved in the oxidative phosphorylation of ATP, providing the body with a steady stream of energy. Once the DNA encoding this kind of protein in mitochondria is disordered, it will lead to reduced energy generation and insufficient ATP supply, which will definitely affect the function of cardiac myocytes and further affect the function of the heart. Mitochondria are especially vital to cardiomyocytes, because mitochondria account for 40% of the size of cardiomyocytes, and 90% of the energy needed for the heart is supplied by mitochondria [20]. In recent years, it has been found that mitochondria are not only an important source of ATP production in cardiomyocytes but also play an important role in cell growth, apoptosis, heat generation and redox signal transduction. Mitochondrial dynamics is involved in processes such as mitochondrial biogenesis, cell metabolism, cell proliferation, cell survival and even cell death[25]. A controlled amount of ROS and calcium can act as a second messenger in the signal transduction of cells and play an important role in maintaining the normal function of the cells[26]. Mitochondria are important organelles for calcium handling/storage, and they play an important role in the control of ROS production and the stability of calcium homeostasis. However, as a result of the lack of effective protective measures and repair mechanisms for the mitochondrial genome, the genome is constantly damaged and deleted, resulting in damage to many functions of the mitochondria itself, such as the production of a large number of ROS. The problems of mitochondrial dynamic fusion and division and the disorder of signal transduction result in cell damage; the corresponding disease is gradually caused by the accumulation of these errors. Cardiovascular disease is characterized by an "aging" mechanism in which function declines over time, much like the process of mitochondrial genome damage[27].

Mitochondrial DNA damage is closely related to mitochondrial transcription proteins and mitochondrial function expression. The mtDNA copy number is not a direct index of an mtDNA damage measurement. It is related to mitochondrial enzyme activity and adenosine triphosphate production, so it can indirectly reflect the function of the mitochondria[28]. Therefore, the mtDNA copy number can be used as a biological indicator of mitochondrial function. The content of mtDNA in white blood cells reflects oxidative induced cell damage that has been widely observed in CVDs[22]. Studies have also shown that there is a positive correlation between mitochondria in peripheral blood and mitochondria in cardiomyocytes[29]. Although it was followed with several limitations, there was clinical significance that the mtDNA copy number in peripheral blood demonstrated potential as a predictor of cardiovascular events. First, the mtDNA copy number has an important predictive value that changes prior to the occurrence of organic and functional changes in the heart, more than other clinical biomarkers such as BNP and NT-proBNP. Additionally, it is convenient and affordable to patients for us to use the mtDNA copy number to detect CVDs, as we only need a small amount of peripheral blood for testing.

The main limitation of our meta-analysis is that the results were pooled from all types of CVD, as there are limited studies among each specific type of CVD. Not all studies included in the meta-analysis adjusted for the same risk factors. Different cohort studies involved different ethnic groups, leading to confounding factors that could affect the end results. Moreover, only 5 studies were included in our meta-analysis, so future research might change the direction of the pooled results. Problems with accuracy and sensitivity with utilizing the mtDNA copy number from SNP microarrays certainly should also be taken as a potential limitation. In addition, all the included studies were cross-sectional in design, which limited the conclusion of the paper to one of association; therefore, better designed longitudinal research needs to be launched to identify the mtDNA copy number as a biomarker for predicting cardiovascular diseases.

## 5. Conclusion

In conclusion, although interstudy variability was present, the performance test of mtDNA for detecting the risk of CVD and SCD revealed that the mtDNA copy number presented the potential to be a biomarker for distinguishing the severity of CVD and potential SCD prediction based on the current data. A lower level of mtDNA indicates a high risk for CVD and an increased risk of SCD. More work should be done to identify whether the mtDNA copy number can be used as a biomarker for CVD risk to help improve the disease prognosis based on a larger sample size and with randomized studies.

## Supporting information

S1 Table PRISMA checklist. (DOC)

S1 File The list of all the excluded article in the analysis. (DOCX)
