# Supplementary material for: Association between mitochondrial DNA copy number and cardiovascular disease: Current evidence based on a systematic review and meta-analysis
Source: PLoS One. 2018 Nov 7;13(11):e0206003. doi: 10.1371/journal.pone.0206003 (PMC6221293; doi:10.1371/journal.pone.0206003)
Supplement: S1 Table — (DOC) [file pone.0206003.s001.doc]

| **Section/topic** | **#** | **Checklist item** | **Reported on page #** |
| --- | --- | --- | --- |
| **TITLE** | | |  |
| Title | 1 | Association between Mitochondrial DNA Copy Number and Cardiovascular Disease: Current Evidence Based on A Systematic Review and Meta-Analysis | 1 |
| **ABSTRACT** | | |  |
| Structured summary | 2 | Background：Mitochondria are energy-producing structure of the cell and help to maintain oxidation stress. In cardiovascular disease, the number of mitochondrial DNA (mtDNA) changes accordingly. Some investigators ask whether it can be used as a new biomarker to predict cardiovascular disease and its adverse events. Thus, we conduct this meta-analysis to assess the role of circulating mtDNA as a biomarker in evaluating cardiovascular disease.  Methods: This meta-analysis was conducted in accordance with a predetermined protocol following the recommendations of Cochrane Handbook of Systematic Reviews. We searched the Pubmed, Embase, the Cochrane Central Register of Controlled Trials and World Health Organization clinical trials registry center to identify relevant studies up to the end of October 2017. Data were analyzed using STATA Version 14.0. Besides, publication bias and meta-regression analysis were also conducted by STATA version 14.0.  Results: We collected results from 5 articles for further analyses. The normalized mtDNA copy number level is lower in cardiovascular disease (CVD) than health control groups with a pooled mean standard deviation (SMD) of -0.36(95%CI,-0.65 to -0.08); The pooled odd ratio (OR) for CVD proportion associated with a 1-SD (standard deviation) decrease in mtDNA copy number level is 1.23( 95% CI,1.06-1.42); The OR for CVD patients with mtDNA copy number lower than median level is 1.88(95% CI,1.65-2.13); The OR for CVD patients with mtDNA copy number located in the lowest quartile part is 2.15(95% CI, 1.46-3.18); the OR between mtDNA copy number and the risk of cardiac sudden death (SCD) is 1.83(95% CI, 1.22-2.74).  Conclusion: Although inter-study variability, the overall performance test of mtDNA for evaluating CVD and SCD revealed that the mtDNA copy number presented the potential to be a biomarker for CVD and SCD prediction. Given that, the fewer copies of mtDNA, the higher the risk of CVD. | 2 |
| **INTRODUCTION** | | |  |
| Rationale | 3 | Cardiovascular diseases (CVD), especially coronary vascular disease, ischemic heart failure, and cardiomyopathy, are major causes of clinical mortality, and lead to be a significant health and economic burden worldwide. Decades past since researchers started to chasing for indicators to predict the prognosis of CVDs. However, although some parameters had been identified, most of them such as BNP, NT-proBNP focused on the acute process of CVDs. Take the advantages of them, diagnosis of specific diseases can be reached, unfortunately they can only provide limited information of real cardiomyocyte function and understanding of their prognosis. Accordingly, it is an emerging demand to find a new and highly specific biomarker to reveal the real cardiac function and predict the prognosis of CVDs. | 3 |
| Objectives | 4 | Association between Mitochondrial DNA Copy Number and Cardiovascular Disease | 3 |
| **METHODS** | | |  |
| Protocol and registration | 5 | This analysis was conducted in accordance with a predetermined protocol following the recommendations of Cochrane Handbook of Systematic Reviews. | 3 |
| Eligibility criteria | 6 | Association between Mitochondrial DNA Copy Number and Cardiovascular Disease. the data collection and reporting were in accordance with Preferred Reporting Items for Systematic Reviews and Meta-Analyses: The PRISMA Statement. The scale of Newcastle-Ottawa Scale (NOS) for assessing the quality of nonrandomized studies in meta-analyses has been taken into evaluating all the included studies. The qualities of enrolled studies were evaluated from 3 parts as selection, comparability and outcome. This meta-analysis would prefer to include the studies deemed moderate to high methodological quality which should be scored at least 5 stars . | 4 |
| Information sources | 7 | We searched the Pubmed, Embase, the Cochrane Central Register of Controlled Trials and World Health Organization clinical trials registry center using a high sensitive and high specific strategy to get the publication which were need to be reviewed, which was “((((copy number) AND ((mitochondrial [MeSH Terms] AND DNA) OR mtDNA OR mitochondrial DNA))) AND ((heart OR cardiology OR cardiac OR cardiology [MeSH Terms] OR heart [MeSH Terms] OR cardiovascular [MeSH Terms]))”. Search was updated to the end of October 2017. | 4 |
| Search | 8 | ((((copy number) AND ((mitochondrial [MeSH Terms] AND DNA) OR mtDNA OR mitochondrial DNA))) AND ((heart OR cardiology OR cardiac OR cardiology [MeSH Terms] OR heart [MeSH Terms] OR cardiovascular [MeSH Terms])) | 4 |
| Study selection | 9 | Citations initially selected by systematic search were first retrieved as title and/or abstract and preliminarily screened. Potentially relevant reports were then retrieved as complete manuscripts and assessed for compliance to inclusion and exclusion criteria.  The inclusion criteria were as followings:  1) Case-control or cohort study design for research study; 2) The association level of mtDNA copy number and CVD’s risks should be evaluated based on case-control or cohort study; 3) Studies should on human beings; 4) Total DNA were extracted from circulating blood sample; 5) MtDNA copy numbers were measured by convinced ways as qPCR or DNA sequence array; 6) All the data should be present as ORs and their 95%CI, or could be converted in to this available data for meta-analysis; 7) All the included studies should be clearly describe how to identify the low level mtDNA copy number and its standard; 8) The data in the publication are sufficient for present  Studies were excluded if any of the following applies:  The exclusion criteria were as followings: 1) Repeat publications, abstracts, letters or reviews; 2) Researches failed to get adjusted OR to reduce the bias; 3) Using mtDNA copy number to tell apart different types of CVD without a sufficient health control population or cohort; 4) research focused on cerebral vascular diseases. | 4 |
| Data collection process | 10 | Two investigators (Peng Yue, Lei Liu) independently assessed eligibility of reports at the title and/or at abstract level, with a third reviewer (Yifei Li) determining the divergences together; studies that meet the inclusion criteria were selected for further analysis. All the baseline data of included studies were extracted which were shown on Table 1. | 4 |
| Data items | 11 | The relative odds ratio (OR) with 95% confidence interval (CI) was used. OR: odds ratio, ESS: effective sample size, SMD: the mean standard deviation. | 5 |
| Risk of bias in individual studies | 12 | Publication bias was tested using funnel plots and the Egger’s test by Stata statistical software (STATA) version 14.0. An asymmetric distribution of data points in the funnel plot and a quantified result of P, 0.05 in the Egger’s test indicated the presence of potential publication bias | 5 |
| Summary measures | 13 | The relative odds ratio (OR) with 95% confidence interval (CI) was used. | 5 |
| Synthesis of results | 14 | Data were analyzed using STATA Version 14.0. Besides, publication bias and meta-regression analysis were conducted by STATA version 14.0. If there was an obvious heterogeneity among the studies (I2 > 50%), the random-effects model was used for the meta-analysis. Otherwise, the fixed-effect model was used. | 5 |

Page 1 of 2

| **Section/topic** | **#** | **Checklist item** | **Reported on page #** |
| --- | --- | --- | --- |
| Risk of bias across studies | 15 | Publication bias was tested using funnel plots and the Egger’s test by Stata statistical software (STATA) version 14.0. An asymmetric distribution of data points in the funnel plot and a quantified result of P, 0.05 in the Egger’s test indicated the presence of potential publication bias. | 5 |
| Additional analyses | 16 | Heterogeneity  The Χ2 test was used to examine heterogeneity in pooling sensitivity and specificity. Heterogeneity was considered to be statistically significant when P＜0.05 in these qualitative tests. The I2 test was also conducted in every pooling analysis to quantitatively estimate the proportion of total variation across studies that was attributable to heterogeneity rather than chance. The I2 value would range from 0 to 100%, with a value over 50% indicating significant heterogeneity.  Sensitivity Analysis  To determine whether any single study was incurring undue weight in the analysis, the sensitivity analysis was conducted for every study using STATA 14.0 for meta-analysis fixed/random-effects estimates.. | 5 |
| **RESULTS** | | |  |
| Study selection | 17 | A total of 132 citations were retrieved. After reading titles and abstracts, 119 citations were excluded according to the selection criteria, and identified the initially 13 articles (2) (8, 12-22). Among them, 8 articles were excluded by reading the completed articles, in which 4 articles were unable to extract useful data for meta-analysis, 2 articles were not focused on the relationship between CVD and mtDNA copy number and 2 articles used mtDNA copy number to tell apart different types of CVD without a sufficient health control population or cohort. Then, none article was added through manual retrospective research after reading related publications. At last 5 articles with 7 studies for mtDNA copy number association with CVD were enrolled into this meta-analysis (Fig 1). Particularly, one article included three studies: Cardiovascular Health Study (CHS), Atherosclerosis Risk in Communities Study (ARIC), and Multiethnic Study of Atherosclerosis (MESA). The basic characteristics of included studies were presented in Table 1. | 6 |
| Study characteristics | 18 | At last 5 articles with 7 studies for mtDNA copy number association with CVD were enrolled into this meta-analysis (Fig 1). Particularly, one article included three studies: Cardiovascular Health Study (CHS), Atherosclerosis Risk in Communities Study (ARIC), and Multiethnic Study of Atherosclerosis (MESA). The basic characteristics of included studies were presented in Table 1. | 6 |
| Risk of bias within studies | 19 | According to Egger’s test, qualitative analysis indicated that the absence of publication bias among all the enrolled studies, for mtDNA copy number, p=0.495, t=-1.02, 95%CI (-63.23, 53.87); for risk in every 1-SD decreased mtDNA level, p=0.937, t=0.09, 95%CI (-13.73, 14.31); for risk in lower than median mtDNA level, p=0.194, t=3.18, 95%CI (-4.26, 7.10); for risk in the lowest quartile part, p=0.264, t=1.37, 95%CI (-4.95, 12.44); for SCD, there were only two studies included, so qualitative analysis for publication bias was unavailable. | 6 |
| Results of individual studies | 20 | 3.3 Average level of mtDNA copy number. 3.4 The association between mtDNA copy number level and the risks of CVD. 3.5 The association between mtDNA copy number and SCD. 3.6 Sensitivity analysis | 6-7 |
| Synthesis of results | 21 | 3.3 Average level of mtDNA copy number. 3.4 The association between mtDNA copy number level and the risks of CVD. 3.5 The association between mtDNA copy number and SCD. 3.6 Sensitivity analysis | 6-7 |
| Risk of bias across studies | 22 | According to Egger’s test, qualitative analysis indicated that the absence of publication bias among all the enrolled studies, for mtDNA copy number, p=0.495, t=-1.02, 95%CI (-63.23, 53.87); for risk in every 1-SD decreased mtDNA level, p=0.937, t=0.09, 95%CI (-13.73, 14.31); for risk in lower than median mtDNA level, p=0.194, t=3.18, 95%CI (-4.26, 7.10); for risk in the lowest quartile part, p=0.264, t=1.37, 95%CI (-4.95, 12.44); for SCD, there were only two studies included, so qualitative analysis for publication bias was unavailable. | 6 |
| Additional analysis | 23 | 3.6 Sensitivity analysis  We systematically qualitatively analyze the sensitivity across included studies (figure 8). The results of figure A, B, C, D and E indicated that no single data set carried enough weight to significantly influence the pooled test performance reported for the ability of the mtDNA copy number in predicting CVD and SCD. Finally, sensitivity analysis had been double checked by removing one data set at a time and the analysis confirmed in both direction and magnitude of statistical significance the findings of the overall analysis | 7 |
| **DISCUSSION** | | |  |
| Summary of evidence | 24 | In this meta-analysis, we conducted three merger evaluations, which are 1) to evaluate the normalized mtDNA copy number level between CVD and health control groups; 2) to evaluate the association between different thresholds of mtDNA copy number and the risks of CVD; and 3) to evaluate the association between mtDNA copy number and the risk of SCD. | 8 |
| Limitations | 25 | The limitation of this meta-analysis is that the results were pooled from all types of CVD as there are limited studies among each specific type of CVD. | 10 |
| Conclusions | 26 | In conclusion, although inter-study variability, the performance test of mtDNA for detecting the risk of CVD and SCD revealed that the mtDNA copy number presented the potential to be a biomarker for distinguishing severe level of CVD and potential SCD prediction. Indicating that, the lower level of mtDNA would announce the CVD with high risk and more possibilities of SCD attacks. And there is still need more works to be done to launch the application of mtDNA copy number as a biomarker for CVD risk level separating in clinic. | 10 |
| **FUNDING** | | |  |
| Funding | 27 | This work was supported by grants from the National Natural Science Foundation of China (No. 81700360, 81741025, 81570369 and 81571515) and the Technology Project of Sichuan Province of China (2016SZ0056). | 10 |

*From:*  Moher D, Liberati A, Tetzlaff J, Altman DG, The PRISMA Group (2009). Preferred Reporting Items for Systematic Reviews and Meta-Analyses: The PRISMA Statement. PLoS Med 6(7): e1000097. doi:10.1371/journal.pmed1000097

For more information, visit: **www.prisma-statement.org**.

Page 2 of 2
