# Supplementary material for: Association between mitochondrial DNA copy number and cardiovascular disease: Current evidence based on a systematic review and meta-analysis
Source: PLoS One. 2018 Nov 7;13(11):e0206003. doi: 10.1371/journal.pone.0206003 (PMC6221293; doi:10.1371/journal.pone.0206003)
Supplement: S1 File — (DOCX) [file pone.0206003.s002.docx]

S1 file.

The list of all the excluded articles (first screen):

^1-121^

1. Zhang SB, Yang S, Zhang Z, et al. Thoracic gamma irradiation-induced obesity in C57BL/6 female mice. *International journal of radiation biology* 2017: 1-9.

2. Ye W, Tang X, Yang Z, et al. Plasma-derived exosomes contribute to inflammation via the TLR9-NF-kappaB pathway in chronic heart failure patients. *Molecular immunology* 2017; **87**: 114-121.

3. Wu B, Ni H, Li J, et al. The Impact of Circulating Mitochondrial DNA on Cardiomyocyte Apoptosis and Myocardial Injury After TLR4 Activation in Experimental Autoimmune Myocarditis. *Cellular physiology and biochemistry : international journal of experimental cellular physiology, biochemistry, and pharmacology* 2017; **42** (2): 713-728.

4. Theeuwes WF, Gosker HR, Langen RCJ, et al. Inactivation of glycogen synthase kinase-3beta (GSK-3beta) enhances skeletal muscle oxidative metabolism. *Biochimica et biophysica acta* 2017; **1863** (12): 3075-3086.

5. Su Z, Klein JD, Du J, et al. Chronic kidney disease induces autophagy leading to dysfunction of mitochondria in skeletal muscle. *American journal of physiology Renal physiology* 2017; **312** (6): F1128-f1140.

6. Lunnon K, Keohane A, Pidsley R, et al. Mitochondrial genes are altered in blood early in Alzheimer's disease. *Neurobiology of aging* 2017; **53**: 36-47.

7. Lee YL, Obiako B, Gorodnya OM, et al. Mitochondrial DNA Damage Initiates Acute Lung Injury and Multi-Organ System Failure Evoked in Rats by Intra-Tracheal Pseudomonas Aeruginosa. *Shock (Augusta, Ga)* 2017; **48** (1): 54-60.

8. Khodambashi Emami N, Golian A, Danesh Mesgaran M, Anthony NB, Rhoads DD. Mitochondrial biogenesis and PGC-1alpha gene expression in male broilers from ascites-susceptible and -resistant lines. *Journal of animal physiology and animal nutrition* 2017.

9. Huang Y, Hong H, Li M, et al. Age-Dependent Oxidative DNA Damage Does Not Correlate with Reduced Proliferation of Cardiomyocytes in Humans. *PloS one* 2017; **12** (1): e0170351.

10. Cui L, Guo J, Zhang Q, et al. Erythropoietin activates SIRT1 to protect human cardiomyocytes against doxorubicin-induced mitochondrial dysfunction and toxicity. *Toxicology letters* 2017; **275**: 28-38.

11. Buck E, Zugel M, Schumann U, et al. High-resolution respirometry of fine-needle muscle biopsies in pre-manifest Huntington's disease expansion mutation carriers shows normal mitochondrial respiratory function. *PloS one* 2017; **12** (4): e0175248.

12. Yuan H, Zhang Q, Guo J, et al. A PGC-1alpha-Mediated Transcriptional Network Maintains Mitochondrial Redox and Bioenergetic Homeostasis against Doxorubicin-Induced Toxicity in Human Cardiomyocytes: Implementation of TT21C. *Toxicological sciences : an official journal of the Society of Toxicology* 2016; **150** (2): 400-17.

13. Wang L, Zhang X, Chan JY, et al. A Novel Danshensu Derivative Prevents Cardiac Dysfunction and Improves the Chemotherapeutic Efficacy of Doxorubicin in Breast Cancer Cells. *Journal of cellular biochemistry* 2016; **117** (1): 94-105.

14. Skarpengland T, Dahl TB, Skjelland M, et al. Enhanced base excision repair capacity in carotid atherosclerosis may protect nuclear DNA but not mitochondrial DNA. *Free radical biology & medicine* 2016; **97**: 386-397.

15. Shukla A, Hebbar M, Harms FL, Kadavigere R, Girisha KM, Kutsche K. Phenotypic variability in patients with interstitial 6q21-q22 microdeletion and Acro-Cardio-Facial syndrome. *American journal of medical genetics Part A* 2016; **170** (11): 2998-3003.

16. Mdaki KS, Larsen TD, Wachal AL, et al. Maternal high-fat diet impairs cardiac function in offspring of diabetic pregnancy through metabolic stress and mitochondrial dysfunction. *American journal of physiology Heart and circulatory physiology* 2016; **310** (6): H681-92.

17. Marin-Garcia J. Mitochondrial DNA repair: a novel therapeutic target for heart failure. *Heart failure reviews* 2016; **21** (5): 475-87.

18. Lipshultz SE, Anderson LM, Miller TL, et al. Impaired mitochondrial function is abrogated by dexrazoxane in doxorubicin-treated childhood acute lymphoblastic leukemia survivors. *Cancer* 2016; **122** (6): 946-53.

19. Inoue T, Ikeda M, Ide T, et al. Twinkle overexpression prevents cardiac rupture after myocardial infarction by alleviating impaired mitochondrial biogenesis. *American journal of physiology Heart and circulatory physiology* 2016; **311** (3): H509-19.

20. Chen YM, Li X, Song GX, et al. Effect of LYRM1 knockdown on proliferation, apoptosis, differentiation and mitochondrial function in the P19 cell model of cardiac differentiation in vitro. *Journal of bioenergetics and biomembranes* 2016; **48** (1): 33-41.

21. Cagnone G, Tsai TS, Srirattana K, et al. Segregation of Naturally Occurring Mitochondrial DNA Variants in a Mini-Pig Model. *Genetics* 2016; **202** (3): 931-44.

22. Zhu S, Hu X, Yu Z, et al. Effect of miR-20b on Apoptosis, Differentiation, the BMP Signaling Pathway and Mitochondrial Function in the P19 Cell Model of Cardiac Differentiation In Vitro. *PloS one* 2015; **10** (4): e0123519.

23. Xie YM, Jin L, Chen XJ, et al. Quantitative changes in mitochondrial DNA copy number in various tissues of pigs during growth. *Genetics and molecular research : GMR* 2015; **14** (1): 1662-70.

24. Tyrka AR, Carpenter LL, Kao HT, et al. Association of telomere length and mitochondrial DNA copy number in a community sample of healthy adults. *Experimental gerontology* 2015; **66**: 17-20.

25. Snowdin JW, Hsiung CH, Kesterson DG, Kamath VG, McKee EE. Effects of Zidovudine Treatment on Heart mRNA Expression and Mitochondrial DNA Copy Number Associated with Alterations in Deoxynucleoside Triphosphate Composition in a Neonatal Rat Model. *Antimicrobial agents and chemotherapy* 2015; **59** (10): 6328-36.

26. Lee SR, Heo HJ, Jeong SH, et al. Low abundance of mitochondrial DNA changes mitochondrial status and renders cells resistant to serum starvation and sodium nitroprusside insult. *Cell biology international* 2015; **39** (7): 865-72.

27. Kang KW, Kim OS, Chin JY, et al. Diastolic Dysfunction Induced by a High-Fat Diet Is Associated with Mitochondrial Abnormality and Adenosine Triphosphate Levels in Rats. *Endocrinology and metabolism (Seoul, Korea)* 2015; **30** (4): 557-68.

28. Ikeda M, Ide T, Fujino T, et al. Overexpression of TFAM or twinkle increases mtDNA copy number and facilitates cardioprotection associated with limited mitochondrial oxidative stress. *PloS one* 2015; **10** (3): e0119687.

29. Guo Q, Guo J, Yang R, et al. Cyclovirobuxine D Attenuates Doxorubicin-Induced Cardiomyopathy by Suppression of Oxidative Damage and Mitochondrial Biogenesis Impairment. *Oxidative medicine and cellular longevity* 2015; **2015**: 151972.

30. Deus CM, Zehowski C, Nordgren K, Wallace KB, Skildum A, Oliveira PJ. Stimulating basal mitochondrial respiration decreases doxorubicin apoptotic signaling in H9c2 cardiomyoblasts. *Toxicology* 2015; **334**: 1-11.

31. Bruns DR, Brown RD, Stenmark KR, Buttrick PM, Walker LA. Mitochondrial integrity in a neonatal bovine model of right ventricular dysfunction. *American journal of physiology Lung cellular and molecular physiology* 2015; **308** (2): L158-67.

32. Bliksoen M, Baysa A, Eide L, et al. Mitochondrial DNA damage and repair during ischemia-reperfusion injury of the heart. *Journal of molecular and cellular cardiology* 2015; **78**: 9-22.

33. Akhmedov AT, Marin-Garcia J. Mitochondrial DNA maintenance: an appraisal. *Molecular and cellular biochemistry* 2015; **409** (1-2): 283-305.

34. Aiken CE, Tarry-Adkins JL, Ozanne SE. Transgenerational Developmental Programming of Ovarian Reserve. *Scientific reports* 2015; **5**: 16175.

35. Qin C, Zhou S, Xiao Y, Chen L. Erythropoietin enhances mitochondrial biogenesis in cardiomyocytes exposed to chronic hypoxia through Akt/eNOS signalling pathway. *Cell biology international* 2014; **38** (3): 335-42.

36. Latouche C, Heywood SE, Henry SL, et al. Maternal overnutrition programs changes in the expression of skeletal muscle genes that are associated with insulin resistance and defects of oxidative phosphorylation in adult male rat offspring. *The Journal of nutrition* 2014; **144** (3): 237-44.

37. Khiati S, Dalla Rosa I, Sourbier C, et al. Mitochondrial topoisomerase I (top1mt) is a novel limiting factor of doxorubicin cardiotoxicity. *Clinical cancer research : an official journal of the American Association for Cancer Research* 2014; **20** (18): 4873-81.

38. Guo J, Guo Q, Fang H, et al. Cardioprotection against doxorubicin by metallothionein Is associated with preservation of mitochondrial biogenesis involving PGC-1alpha pathway. *European journal of pharmacology* 2014; **737**: 117-24.

39. Garrett SM, Whitaker RM, Beeson CC, Schnellmann RG. Agonism of the 5-hydroxytryptamine 1F receptor promotes mitochondrial biogenesis and recovery from acute kidney injury. *The Journal of pharmacology and experimental therapeutics* 2014; **350** (2): 257-64.

40. Chen Y, Sparks M, Bhandari P, Matkovich SJ, Dorn GW, 2nd. Mitochondrial genome linearization is a causative factor for cardiomyopathy in mice and Drosophila. *Antioxidants & redox signaling* 2014; **21** (14): 1949-59.

41. Tanaka A, Ide T, Fujino T, et al. The overexpression of Twinkle helicase ameliorates the progression of cardiac fibrosis and heart failure in pressure overload model in mice. *PloS one* 2013; **8** (6): e67642.

42. Sun M, Huang C, Wang C, et al. Ginsenoside Rg3 improves cardiac mitochondrial population quality: mimetic exercise training. *Biochemical and biophysical research communications* 2013; **441** (1): 169-74.

43. Sun L, Zhao M, Yu XJ, et al. Cardioprotection by acetylcholine: a novel mechanism via mitochondrial biogenesis and function involving the PGC-1alpha pathway. *Journal of cellular physiology* 2013; **228** (6): 1238-48.

44. Samuels DC, Han L, Li J, et al. Finding the lost treasures in exome sequencing data. *Trends in genetics : TIG* 2013; **29** (10): 593-9.

45. Pohjoismaki JL, Kruger M, Al-Furoukh N, Lagerstedt A, Karhunen PJ, Braun T. Postnatal cardiomyocyte growth and mitochondrial reorganization cause multiple changes in the proteome of human cardiomyocytes. *Molecular bioSystems* 2013; **9** (6): 1210-9.

46. Miao Y, Zhou J, Zhao M, et al. Acetylcholine attenuates hypoxia/ reoxygenation-induced mitochondrial and cytosolic ROS formation in H9c2 cells via M2 acetylcholine receptor. *Cellular physiology and biochemistry : international journal of experimental cellular physiology, biochemistry, and pharmacology* 2013; **31** (2-3): 189-98.

47. Liu YQ, Song GX, Liu HL, et al. Silencing of FABP3 leads to apoptosis-induced mitochondrial dysfunction and stimulates Wnt signaling in zebrafish. *Molecular medicine reports* 2013; **8** (3): 806-12.

48. Liu J, Lloyd SG. High-fat, low-carbohydrate diet alters myocardial oxidative stress and impairs recovery of cardiac function after ischemia and reperfusion in obese rats. *Nutrition research (New York, NY)* 2013; **33** (4): 311-21.

49. Kumarasamy S, Gopalakrishnan K, Abdul-Majeed S, Partow-Navid R, Farms P, Joe B. Construction of two novel reciprocal conplastic rat strains and characterization of cardiac mitochondria. *American journal of physiology Heart and circulatory physiology* 2013; **304** (1): H22-32.

50. Finsterer J, Stollberger C. Mitochondrial DNA sequence and copy number variants cannot be the only culprit in the pathogenesis of noncompaction. *Molecular genetics and metabolism* 2013; **109** (3): 317.

51. Aiken CE, Tarry-Adkins JL, Ozanne SE. Suboptimal nutrition in utero causes DNA damage and accelerated aging of the female reproductive tract. *FASEB journal : official publication of the Federation of American Societies for Experimental Biology* 2013; **27** (10): 3959-65.

52. Wills LP, Trager RE, Beeson GC, et al. The beta2-adrenoceptor agonist formoterol stimulates mitochondrial biogenesis. *The Journal of pharmacology and experimental therapeutics* 2012; **342** (1): 106-18.

53. Fujino T, Ide T, Yoshida M, et al. Recombinant mitochondrial transcription factor A protein inhibits nuclear factor of activated T cells signaling and attenuates pathological hypertrophy of cardiac myocytes. *Mitochondrion* 2012; **12** (4): 449-58.

54. Chen L, Liu T, Tran A, et al. OPA1 mutation and late-onset cardiomyopathy: mitochondrial dysfunction and mtDNA instability. *Journal of the American Heart Association* 2012; **1** (5): e003012.

55. Watanabe A, Arai M, Koitabashi N, et al. Mitochondrial transcription factors TFAM and TFB2M regulate Serca2 gene transcription. *Cardiovascular research* 2011; **90** (1): 57-67.

56. Tornatore TF, Dalla Costa AP, Clemente CF, et al. A role for focal adhesion kinase in cardiac mitochondrial biogenesis induced by mechanical stress. *American journal of physiology Heart and circulatory physiology* 2011; **300** (3): H902-12.

57. Muller-Hocker J, Horvath R, Schafer S, et al. Mitochondrial DNA depletion and fatal infantile hepatic failure due to mutations in the mitochondrial polymerase gamma (POLG) gene: a combined morphological/enzyme histochemical and immunocytochemical/biochemical and molecular genetic study. *Journal of cellular and molecular medicine* 2011; **15** (2): 445-56.

58. Liu J, Wang P, Luo J, et al. Peroxisome proliferator-activated receptor beta/delta activation in adult hearts facilitates mitochondrial function and cardiac performance under pressure-overload condition. *Hypertension (Dallas, Tex : 1979)* 2011; **57** (2): 223-30.

59. Guo J, Zheng L, Liu W, et al. Frequent truncating mutation of TFAM induces mitochondrial DNA depletion and apoptotic resistance in microsatellite-unstable colorectal cancer. *Cancer research* 2011; **71** (8): 2978-87.

60. Alan L, Spacek T, Zelenka J, et al. Assessment of mitochondrial DNA as an indicator of islet quality: an example in Goto Kakizaki rats. *Transplantation proceedings* 2011; **43** (9): 3281-4.

61. Wang P, Liu J, Li Y, et al. Peroxisome proliferator-activated receptor {delta} is an essential transcriptional regulator for mitochondrial protection and biogenesis in adult heart. *Circulation research* 2010; **106** (5): 911-9.

62. Pohjoismaki JL, Goffart S, Taylor RW, et al. Developmental and pathological changes in the human cardiac muscle mitochondrial DNA organization, replication and copy number. *PloS one* 2010; **5** (5): e10426.

63. Carraway MS, Suliman HB, Jones WS, Chen CW, Babiker A, Piantadosi CA. Erythropoietin activates mitochondrial biogenesis and couples red cell mass to mitochondrial mass in the heart. *Circulation research* 2010; **106** (11): 1722-30.

64. Brinckmann A, Weiss C, Wilbert F, et al. Regionalized pathology correlates with augmentation of mtDNA copy numbers in a patient with myoclonic epilepsy with ragged-red fibers (MERRF-syndrome). *PloS one* 2010; **5** (10): e13513.

65. Balcarek K, Venhoff N, Deveaud C, et al. Role of pyrimidine depletion in the mitochondrial cardiotoxicity of nucleoside analogue reverse transcriptase inhibitors. *Journal of acquired immune deficiency syndromes (1999)* 2010; **55** (5): 550-7.

66. Zecic A, Smet JE, De Praeter CM, et al. Lactic acidosis in a newborn with adrenal calcifications. *Pediatric research* 2009; **66** (3): 317-22.

67. van der Kraan PM, Blaney Davidson EN, Blom A, van den Berg WB. TGF-beta signaling in chondrocyte terminal differentiation and osteoarthritis: modulation and integration of signaling pathways through receptor-Smads. *Osteoarthritis and cartilage* 2009; **17** (12): 1539-45.

68. Rhodes MA, Carraway MS, Piantadosi CA, et al. Carbon monoxide, skeletal muscle oxidative stress, and mitochondrial biogenesis in humans. *American journal of physiology Heart and circulatory physiology* 2009; **297** (1): H392-9.

69. Reynolds CM, Suliman HB, Hollingsworth JW, Welty-Wolf KE, Carraway MS, Piantadosi CA. Nitric oxide synthase-2 induction optimizes cardiac mitochondrial biogenesis after endotoxemia. *Free radical biology & medicine* 2009; **46** (5): 564-72.

70. Miller F, Nagley P, Mariani JA, et al. Age-related decline in stress responses of human myocardium may not be explained by changes in mtDNA. *Mechanisms of ageing and development* 2009; **130** (11-12): 742-7.

71. Lancel S, Hassoun SM, Favory R, Decoster B, Motterlini R, Neviere R. Carbon monoxide rescues mice from lethal sepsis by supporting mitochondrial energetic metabolism and activating mitochondrial biogenesis. *The Journal of pharmacology and experimental therapeutics* 2009; **329** (2): 641-8.

72. Lamperti C, Zeviani M. Encephalomyopathies caused by abnormal nuclear-mitochondrial intergenomic cross-talk. *Acta myologica : myopathies and cardiomyopathies : official journal of the Mediterranean Society of Myology* 2009; **28** (1): 2-11.

73. Chen DD, Dong YG, Liu D, He JG. Epigallocatechin-3-gallate attenuates cardiac hypertrophy in hypertensive rats in part by modulation of mitogen-activated protein kinase signals. *Clinical and experimental pharmacology & physiology* 2009; **36** (9): 925-32.

74. Atig RK, Hsouna S, Beraud-Colomb E, Abdelhak S. [Mitochondrial DNA: properties and applications]. *Archives de l'Institut Pasteur de Tunis* 2009; **86** (1-4): 3-14.

75. Urschel MR, O'Brien KM. High mitochondrial densities in the hearts of Antarctic icefishes are maintained by an increase in mitochondrial size rather than mitochondrial biogenesis. *The Journal of experimental biology* 2008; **211** (Pt 16): 2638-46.

76. Tsutsui H, Kinugawa S, Matsushima S. Oxidative stress and mitochondrial DNA damage in heart failure. *Circulation journal : official journal of the Japanese Circulation Society* 2008; **72 Suppl A**: A31-7.

77. Suarez J, Hu Y, Makino A, Fricovsky E, Wang H, Dillmann WH. Alterations in mitochondrial function and cytosolic calcium induced by hyperglycemia are restored by mitochondrial transcription factor A in cardiomyocytes. *American journal of physiology Cell physiology* 2008; **295** (6): C1561-8.

78. Chen CS, Hiura Y, Shen CS, Iwai N. Assessment of mitochondrial DNA polymorphisms in salt-sensitive hypertension in Dahl salt-sensitive rats. *Hypertension research : official journal of the Japanese Society of Hypertension* 2008; **31** (1): 107-15.

79. Suliman HB, Carraway MS, Tatro LG, Piantadosi CA. A new activating role for CO in cardiac mitochondrial biogenesis. *Journal of cell science* 2007; **120** (Pt 2): 299-308.

80. Lebrecht D, Geist A, Ketelsen UP, Haberstroh J, Setzer B, Walker UA. Dexrazoxane prevents doxorubicin-induced long-term cardiotoxicity and protects myocardial mitochondria from genetic and functional lesions in rats. *British journal of pharmacology* 2007; **151** (6): 771-8.

81. Dong F, Li Q, Sreejayan N, Nunn JM, Ren J. Metallothionein prevents high-fat diet induced cardiac contractile dysfunction: role of peroxisome proliferator activated receptor gamma coactivator 1alpha and mitochondrial biogenesis. *Diabetes* 2007; **56** (9): 2201-12.

82. Chan SS, Santos JH, Meyer JN, et al. Mitochondrial toxicity in hearts of CD-1 mice following perinatal exposure to AZT, 3TC, or AZT/3TC in combination. *Environmental and molecular mutagenesis* 2007; **48** (3-4): 190-200.

83. Blaney Davidson EN, van der Kraan PM, van den Berg WB. TGF-beta and osteoarthritis. *Osteoarthritis and cartilage* 2007; **15** (6): 597-604.

84. Tsutsui H, Ide T, Kinugawa S. Mitochondrial oxidative stress, DNA damage, and heart failure. *Antioxidants & redox signaling* 2006; **8** (9-10): 1737-44.

85. Tsutsui H. Mitochondrial oxidative stress and heart failure. *Internal medicine (Tokyo, Japan)* 2006; **45** (13): 809-13.

86. Taivassalo T, Gardner JL, Taylor RW, et al. Endurance training and detraining in mitochondrial myopathies due to single large-scale mtDNA deletions. *Brain : a journal of neurology* 2006; **129** (Pt 12): 3391-401.

87. St John JC, Amaral A, Bowles E, et al. The analysis of mitochondria and mitochondrial DNA in human embryonic stem cells. *Methods in molecular biology (Clifton, NJ)* 2006; **331**: 347-74.

88. Matsushima S, Ide T, Yamato M, et al. Overexpression of mitochondrial peroxiredoxin-3 prevents left ventricular remodeling and failure after myocardial infarction in mice. *Circulation* 2006; **113** (14): 1779-86.

89. Hayashi M, Imanaka-Yoshida K, Yoshida T, et al. A crucial role of mitochondrial Hsp40 in preventing dilated cardiomyopathy. *Nature medicine* 2006; **12** (1): 128-32.

90. van den Bosch BJ, van den Burg CM, Schoonderwoerd K, et al. Regional absence of mitochondria causing energy depletion in the myocardium of muscle LIM protein knockout mice. *Cardiovascular research* 2005; **65** (2): 411-8.

91. Masuyama M, Iida R, Takatsuka H, Yasuda T, Matsuki T. Quantitative change in mitochondrial DNA content in various mouse tissues during aging. *Biochimica et biophysica acta* 2005; **1723** (1-3): 302-8.

92. Ikeuchi M, Matsusaka H, Kang D, et al. Overexpression of mitochondrial transcription factor a ameliorates mitochondrial deficiencies and cardiac failure after myocardial infarction. *Circulation* 2005; **112** (5): 683-90.

93. Frahm T, Mohamed SA, Bruse P, Gemund C, Oehmichen M, Meissner C. Lack of age-related increase of mitochondrial DNA amount in brain, skeletal muscle and human heart. *Mechanisms of ageing and development* 2005; **126** (11): 1192-200.

94. Tyynismaa H, Sembongi H, Bokori-Brown M, et al. Twinkle helicase is essential for mtDNA maintenance and regulates mtDNA copy number. *Human molecular genetics* 2004; **13** (24): 3219-27.

95. Suliman HB, Welty-Wolf KE, Carraway M, Tatro L, Piantadosi CA. Lipopolysaccharide induces oxidative cardiac mitochondrial damage and biogenesis. *Cardiovascular research* 2004; **64** (2): 279-88.

96. Shen X, Zheng S, Thongboonkerd V, et al. Cardiac mitochondrial damage and biogenesis in a chronic model of type 1 diabetes. *American journal of physiology Endocrinology and metabolism* 2004; **287** (5): E896-905.

97. Rosenfeldt F, Miller F, Nagley P, et al. Response of the senescent heart to stress: clinical therapeutic strategies and quest for mitochondrial predictors of biological age. *Annals of the New York Academy of Sciences* 2004; **1019**: 78-84.

98. Maarouf N, Arno G, Carter ND, et al. Quantification of mitochondrial sublimons in human fibrillating atria. *Clinical science (London, England : 1979)* 2004; **106** (6): 653-9.

99. Suematsu N, Tsutsui H, Wen J, et al. Oxidative stress mediates tumor necrosis factor-alpha-induced mitochondrial DNA damage and dysfunction in cardiac myocytes. *Circulation* 2003; **107** (10): 1418-23.

100. Miller FJ, Rosenfeldt FL, Zhang C, Linnane AW, Nagley P. Precise determination of mitochondrial DNA copy number in human skeletal and cardiac muscle by a PCR-based assay: lack of change of copy number with age. *Nucleic acids research* 2003; **31** (11): e61.

101. Garnier A, Fortin D, Delomenie C, Momken I, Veksler V, Ventura-Clapier R. Depressed mitochondrial transcription factors and oxidative capacity in rat failing cardiac and skeletal muscles. *The Journal of physiology* 2003; **551** (Pt 2): 491-501.

102. Limongelli A, Tiranti V. Inherited Mendelian defects of nuclear-mitochondrial communication affecting the stability of mitochondrial DNA. *Mitochondrion* 2002; **2** (1-2): 39-46.

103. Ide T, Tsutsui H, Hayashidani S, et al. Mitochondrial DNA damage and dysfunction associated with oxidative stress in failing hearts after myocardial infarction. *Circulation research* 2001; **88** (5): 529-35.

104. Barazzoni R, Nair KS. Changes in uncoupling protein-2 and -3 expression in aging rat skeletal muscle, liver, and heart. *American journal of physiology Endocrinology and metabolism* 2001; **280** (3): E413-9.

105. Kopsidas G, Kovalenko SA, Heffernan DR, et al. Tissue mitochondrial DNA changes. A stochastic system. *Annals of the New York Academy of Sciences* 2000; **908**: 226-43.

106. Barazzoni R, Short KR, Nair KS. Effects of aging on mitochondrial DNA copy number and cytochrome c oxidase gene expression in rat skeletal muscle, liver, and heart. *The Journal of biological chemistry* 2000; **275** (5): 3343-7.

107. Marin-Garcia J, Ananthakrishnan R, Goldenthal MJ, Filiano JJ, Perez-Atayde A. Mitochondrial dysfunction in skeletal muscle of children with cardiomyopathy. *Pediatrics* 1999; **103** (2): 456-9.

108. Schultz RA, Swoap SJ, McDaniel LD, et al. Differential expression of mitochondrial DNA replication factors in mammalian tissues. *The Journal of biological chemistry* 1998; **273** (6): 3447-51.

109. Marin-Garcia J, Ananthakrishnan R, Goldenthal MJ. Hypertrophic cardiomyopathy with mitochondrial DNA depletion and respiratory enzyme defects. *Pediatric cardiology* 1998; **19** (3): 266-8.

110. Marin-Garcia J, Ananthakrishnan R, Goldenthal MJ. Human mitochondrial function during cardiac growth and development. *Molecular and cellular biochemistry* 1998; **179** (1-2): 21-6.

111. Larsson NG, Wang J, Wilhelmsson H, et al. Mitochondrial transcription factor A is necessary for mtDNA maintenance and embryogenesis in mice. *Nature genetics* 1998; **18** (3): 231-6.

112. Palmeira CM, Serrano J, Kuehl DW, Wallace KB. Preferential oxidation of cardiac mitochondrial DNA following acute intoxication with doxorubicin. *Biochimica et biophysica acta* 1997; **1321** (2): 101-6.

113. Marin-Garcia J, Ananthakrishnan R, Goldenthal MJ. Mitochondrial gene expression in rat heart and liver during growth and development. *Biochemistry and cell biology = Biochimie et biologie cellulaire* 1997; **75** (2): 137-42.

114. Marin-Garcia J, Ananthakrishnan R, Pierpont ME, Goldenthal MJ. Mitochondrial dysfunction in spontaneous inbred turkey cardiomyopathy. *Biochemistry and molecular biology international* 1996; **38** (6): 1087-93.

115. Marin-Garcia J, Goldenthal MJ, Pierpont ME, Ananthakrishnan R. Impaired mitochondrial function in idiopathic dilated cardiomyopathy: biochemical and molecular analysis. *Journal of cardiac failure* 1995; **1** (4): 285-91.

116. Marin-Garcia J, Goldenthal MJ, Ananthakrishnan R, Mirvis D. Localized mitochondrial dysfunction in canine myocardial ischemia. *Biochemistry and molecular biology international* 1995; **35** (3): 651-9.

117. Marin-Garcia J, Ananthakrishnan R, Goldenthal MJ. Heart mitochondria response to alcohol is different than brain and liver. *Alcoholism, clinical and experimental research* 1995; **19** (6): 1463-6.

118. Gadaleta MN, Rainaldi G, Lezza AM, Milella F, Fracasso F, Cantatore P. Mitochondrial DNA copy number and mitochondrial DNA deletion in adult and senescent rats. *Mutation research* 1992; **275** (3-6): 181-93.

119. Veltri KL, Espiritu M, Singh G. Distinct genomic copy number in mitochondria of different mammalian organs. *Journal of cellular physiology* 1990; **143** (1): 160-4.

120. Annex BH, Williams RS. Mitochondrial DNA structure and expression in specialized subtypes of mammalian striated muscle. *Molecular and cellular biology* 1990; **10** (11): 5671-8.

121. Kuznetsova T, Knez J. Peripheral Blood Mitochondrial DNA and Myocardial Function. *Advances in experimental medicine and biology* 2017; **982**: 347-358.

Excluded articles (Full text screen):

^1-8^

1. Guyatt AL, Burrows K, Guthrie PAI, et al. Cardiometabolic phenotypes and mitochondrial DNA copy number in two cohorts of UK women. *Mitochondrion* 2017.

2. Knez J, Cauwenberghs N, Thijs L, et al. Association of left ventricular structure and function with peripheral blood mitochondrial DNA content in a general population. *International journal of cardiology* 2016; **214**: 180-8.

3. Hernandez-Rios R, Hernandez-Estrada S, Cruz-Robles D, et al. Low fructose and low salt diets increase mitochondrial DNA in white blood cells of overweight subjects. *Experimental and clinical endocrinology & diabetes : official journal, German Society of Endocrinology [and] German Diabetes Association* 2013; **121** (9): 535-8.

4. Ahuja P, Wanagat J, Wang Z, et al. Divergent mitochondrial biogenesis responses in human cardiomyopathy. *Circulation* 2013; **127** (19): 1957-67.

5. Xiao J, Chen L, Wang X, Liu M, Xiao Y. eNOS correlates with mitochondrial biogenesis in hearts of congenital heart disease with cyanosis. *Arquivos brasileiros de cardiologia* 2012; **99** (3): 780-8.

6. Tsutsui H, Kinugawa S, Matsushima S. Mitochondrial oxidative stress and dysfunction in myocardial remodelling. *Cardiovascular research* 2009; **81** (3): 449-56.

7. Mohamed SA, Hanke T, Erasmi AW, et al. Mitochondrial DNA deletions and the aging heart. *Experimental gerontology* 2006; **41** (5): 508-17.

8. Bornstein B, Huertas R, Ochoa P, et al. Mitochondrial gene expression and respiratory enzyme activities in cardiac diseases. *Biochimica et biophysica acta* 1998; **1406** (1): 85-90.
